# Risk factors for failed back surgery syndrome following open posterior lumbar surgery for degenerative lumbar disease

**DOI:** 10.1186/s12891-022-06066-2

**Published:** 2022-12-31

**Authors:** Wenbo Xu, Bingbing Ran, Jianhui Zhao, Wenqi Luo, Rui Gu

**Affiliations:** 1 Departments of Orthopedics, China-Japan Union Hospital of Jilin University, No.126 Xiantai Street, Changchun, 130033 Jilin P.R. China; 2 Departments of Medical Cosmetology, Jilin Province People’s Hospital, No. 1183, Gongnong Road, Changchun, 130021 Jilin P.R. China

**Keywords:** Failed back surgery syndrome, Degenerative lumbar disease, Patient satisfaction, Risk factors, Lumbar spine surgery

## Abstract

**Background:**

With the growing number of traditional posterior open surgery, the incidence of failed back surgery syndrome (FBSS) increases gradually. We aimed to investigate the incidence and risk factors for FBSS following open posterior lumbar surgery for degenerative lumbar disease (DLD).

**Method:**

A multivariable regression analysis was performed for 333 consecutive patients to identify potential risk factors for FBSS. Clinical outcomes were evaluated by the validated North American Spine Society (NASS) Questionnaire and numerical rating scale (NRS) for pain. Demographics, diagnostic characteristics, surgical data, radiographic parameters for each patient were analyzed.

**Result:**

16.8% of the included patients were classified as FBSS. Univariate analysis showed that age, hypertension, symptom location, intermittent claudication, preoperative pain NRS-leg, HIZ, Modic changes (MCs), surgical strategy and postoperative rehabilitation were related to FBSS. Multivariable logistic regression analysis demonstrated that preoperative NRS-leg (OR:0.80, 95%CI:0.71–0.91, *P* = 0.001), hypertension (OR: 2.22, 95%CI: 1.10–4.51, *P* = 0.027), intermittent claudication with waking distance > 100 m (OR: 4.07, 95%CI: 1.75–9.47, *P* = 0.001) and waking distance ≤ 100 m (OR: 12.43, 95%CI: 5.54–27.92, *P* < 0.001), HIZ (OR: 8.26, 95%CI: 4.00–17.04, *P* < 0.001), MCs (OR: 3.41, 95%CI: 1.73–6.71, *P* < 0.001), postoperative rehabilitation (OR: 2.63, 95%CI: 1.13–6.12, *P* = 0.024) were risk factors for FBSS.

**Conclusion:**

Open posterior lumbar surgery is an effective treatment for DLD which provides pain reduction and lumbar curve improvement with a considerable satisfaction rate. Lower preoperative NRS-leg, hypertension, intermittent claudication, HIZ, MCs and postoperative rehabilitation are risk factors for FBSS, which can serve as a tool for clinicians to identify at-risk population and provide more effective management to mitigate the doctor-patient contradictions and further occupation of medical resources.

## Background

In the past three decades, low back pain (LBP) has been the main cause of non-fatal health loss, and the resulting burden poses a severe challenge to the coping ability of health care systems in various countries [1]. Degenerative lumbar disease (DLD) is the main cause of LBP, whose prevalence is positively correlated with the increase of age. With the aggravation of population aging, more and more attention has been paid to this very disease [2, 3].

Traditional posterior open surgery is a classic and effective surgical method for DLD. Nonetheless, as the sophistication of lumbar surgery technology and the increasing number of its implementation, more and more researchers have realized that although the structural deficits of the initial operation have been excluded, postoperative persistent pain and/or numbness in the back or legs still afflict the patients, which results in their dissatisfaction. On the other hand, some patients are still not satisfied even though they have achieved clinical improvement in disability or pain [4].

Failed back Surgery syndrome (FBSS) is a term used to describe patients' dissatisfaction with the effect of lumbar surgery [5]. Over the 40 years since this concept was proposed, the definition of FBSS has been gradually diluted and generalized. A large number of terms have emerged to describe the same condition [6, 7], which has derived heterogeneous diagnostic criteria. (Table 1).

**Table 1:** A summary of diagnostic criteria and characteristics of FBSS

Study	Diagnostic criteria	Characteristics
Merskey et al. [8]	Lumbar (cervical) pain of unknown origin either persisting despite surgical intervention or appearing after surgical intervention for spinal (origin) pain originally in the same topographical distribution	-Unknown origin
Leveque et al. [9]	Chronic radicular pain that has recurred or persists in the same distribution despite anatomically satisfactory previous spinal surgery	-Recurred or persists -Anatomically satisfactory surgery
Waguespack et al. [10]	The outcome of lumbar spine surgery does not meet the pre-surgical expectations of the patient and surgeon	-Didn’t meet the pre-surgical expectations
Kumar et al. [11]	Patients suffer from chronic neuropathic pain with a mean leg pain intensity of > 5 cm on a Visual Analog Scale (VAS) from 0 to 10 cm following anatomically successful spinal surgery	-Anatomically satisfactory surgery -VAS > 5/10
Bokov et al. [12]	Patients with a pain intensity of no less than 40 on the 100-point VAS and at least a 40% decrease on the Oswestry Disability Index (ODI)	-VAS > 40/100 -ODI decrease < 40%
Bordoni et al. [5]	Patient who underwent spinal surgery, irrespective of type or intervention area, with persistent pain in the lumbosacral region with or without it radiating to the leg despite the intervention for up to 3 months	-The duration of symptoms > 3 months and conservative treatment was ineffective
Andres et al. [13]	Patients with chronic, intractable pain (> 5/10 on Numeric Rating Scale) of the trunk and/or limbs that has remained refractory to conservative therapy for at least 6 months	-The duration of symptoms > 6 months -NRS > 5/10
Cho et al. [14]	Patients show chronic back pain or leg pain after successfully performed lumbar surgery without specific reasons such as compressive lesions, infection	-

A large number of studies have been conducted on the risk factors of FBSS, and psychiatric disorders such as depression and insomnia have been proved to be associated with FBSS [15, 16]. To the best of our knowledge, however, there is no research on the relationship between FBSS and the inherent characteristics of DLD, including but not limited to symptoms distribution, degree of intermittent claudication, radiographic parameters and more. It is also unclear whether postoperative rehabilitation affects the incidence of FBSS.

Here, in order to explore the risk factors of FBSS after traditional posterior open surgery, we put forward a more comprehensive diagnostic criterion built on the existing literature and conducted a retrospective study based on prospectively collected data. We hope that this study will increase clinicians' understanding of FBSS and have a positive impact on its management.

## Methods

### Study design and participants

This was a retrospective review of prospectively collected data which incorporated DLD patients who underwent traditional posterior open surgery between January 2018 and August 2020 with a minimum of 3-month follow-up at a single institution. Traditional posterior open surgery, which refers to fenestration discectomy (FD), posterior lumbar interbody fusion (PLIF) and posterolateral fusion (PLF) was performed by 6 senior spine surgeons in our institution. As for the surgical procedures, briefly, all patients were positioned prone under general anesthesia. After the exposure of the spinous processes, laminae, and transverse processes, pedicle screw instrumentation and posterior decompression were carried out for PLF and PLIF groups. In the PLF group, autologous bone from the spinous process and laminae was placed on decorticated transverse processes and facet joints bilaterally. In the PLIF group, the cage with autogenous bone was implanted into the intervertebral space after a discectomy and endplate decortication. For FD, posterior decompression was performed after the removal of the lower edge of the upper laminae.

The inclusion criteria were: (1) the patients were older than 18 years; (2) the diagnosis of DLD, including lumbar disc herniation (LDH), lumbar spinal stenosis (LSS), degenerative spondylolisthesis (Meyerding I-II) was confirmed by X-ray photographs, computer tomography (CT) and magnetic resonance imaging (MRI). (3) all patients met the surgical indication of DLD. Patients who met the following criteria were excluded: (1) definite iatrogenic injury; (2) severe or progressive psychotic disorders; (3) an active workman’s compensation claim or medical disputes; (4) lumbar surgical history; (5) presence of trauma, neoplasm, infection, congenital deformations.

Here, a more quantitative and systematic definition of FBSS was developed as follows: An intractable pain or sensory deficits of low back and/or limbs postoperatively that have remained refractory to conservative treatment (> 3 months), which led to a dissatisfaction with the outcome of the surgery [17]. As for satisfaction, this research adopted the related questions in the validated North American Spine Society (NASS) questionnaire [18] in the form of telephone follow-up to evaluate.

### Clinical and radiological outcomes

The following data were collected preoperatively: age, gender, diagnosis, chronic comorbidity such as hypertension and diabetes mellitus. The location of symptoms (low back pain without lower extremity symptoms, unilateral or bilateral lower extremity symptoms), duration of symptoms, concomitant intermittent claudication (IC), history of exacerbation, history of long-term analgesic use (≥ 6 months), preoperative and postoperative Numerical Rating Scale (NRS)-back and NRS-leg were also analyzed in this study. NRS is an assessment instrument on a scale ranging from 0 to 10, where 0 represented “no pain” and 10 represented “extreme pain”.

**Fig. 1:**
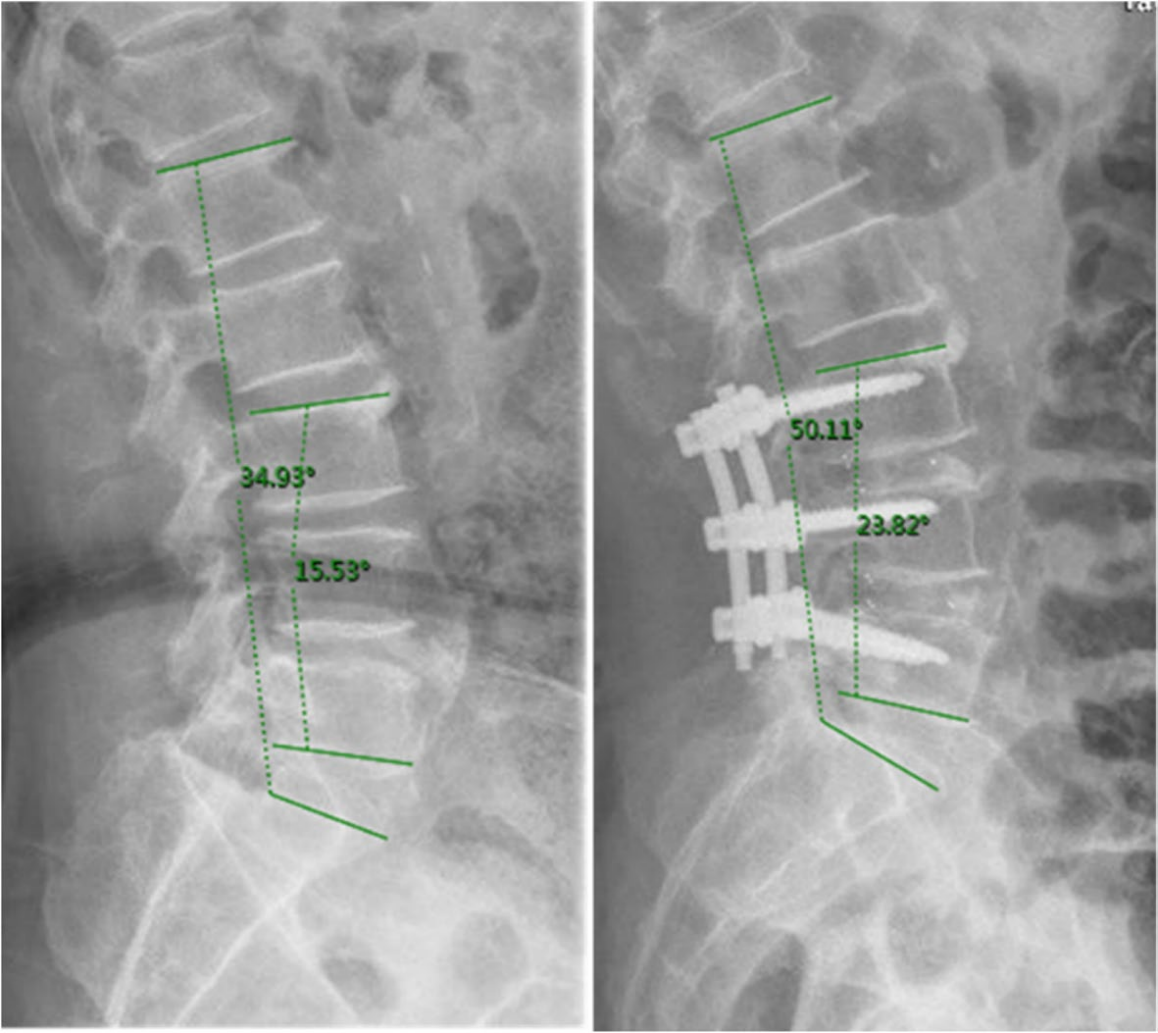
The measurement of lumbar lordosis and segmental lordosis at pre-operation and immediate post-operation

The following two categorized parameters were also analyzed. Surgical data: surgical strategy, surgical segment, operation duration and postoperative rehabilitation. Postoperative rehabilitation refers to the treatment under the guidance of physiotherapist rather than self-management. Radiographic outcomes: high intensity zone (HIZ) and Modic changes (MCs) in MRI. Lumbar lordosis (the Cobb angle between L1 vertebral superior endplate and S1 vertebral superior endplate) and segmental lordosis (the Cobb angle between the superior endplate of the superior vertebra and the inferior endplate of the inferior vertebra) (Fig. 1) [53] were measured at pre-operation and immediate post-operation [19]. To avoid bias, all information was collected by a study personnel unrelated to the operation.

### Statistics

Continuous data are expressed as mean ± standard deviation and categorical data are given as frequency and/or percentage. Independent or paired sample t-test was performed on the data subject to normal distribution, for those who don’t obey normal distribution, Mann–Whitney U test was conducted. Categorical data between two groups were analyzed using the Fisher exact test or chi-squared test, as appropriate. Covariate selection for the multivariable analysis (backward elimination, conditional) was based on *P* < 0.05 in univariate analysis. SPSS version 26.0 software (IBM Corporation, USA) was used to analyze the data. *P* < 0.05 was considered as being statistically significant.

## Results

A total of 333 patients were enrolled in this study. All clinical outcome scores and radiographic parameters were significantly improved postoperatively (*P* < 0.001). (Table 2) 56 patients meeting the inclusion criteria were regarded as the FBSS + group with an incidence rate of 16.8%, whose mean follow-up time was 24.4 ± 6.8 months. The counterpart was 22.4 ± 6.9 months in FBSS- group. The final follow-up rate was 92% and 95% respectively. Reasons of dissatisfaction were revealed through further follow-up: 40 people suffered from persistent postoperative pain, 9 people afflicted by unrelieved or even aggravated numbness, 2 people due to no improvement in lower extremity muscle strength. 2 patients with pain and numbness at the same time and 3 patients with pain accompanied by lower limb weakness.

**Table 2:** Clinical outcome and radiographic parameters

	Pre-operation	Post-operation	*P* value
NRS-Back pain	6.3 ± 2.8	1.6 ± 1.9	< 0.001
NRS-Leg pain	7.2 ± 2.3	0.8 ± 1.6	< 0.001
Lumbar lordosis (°)	31.6 ± 13.8	38.4 ± 11.5	< 0.001
Segmental lordosis (°)	15.6 ± 9.4	20.5 ± 9.5	< 0.001

Demographics of the patients was shown in Table 3. The mean age of FBSS + group and FBSS- group was 60.6 years and 53.8 years respectfully. Patients with hypertension accounted for 64.7% in FBSS + group, the counterpart was 22.6% in FBSS- group. Univariate analysis showed that the age of patients suffered from FBSS was significantly older than those who didn’t (*P* < 0.05). The comorbidity of hypertension in the FBSS + group was more common than that in the FBSS- group (*P* < 0.05). There was no significant difference in gender, diabetes mellitus and diagnosis between the two groups.

**Table 3:** Demographics

	FBSS + (*n* = 56)	FBSS-(*n* = 277)	*P* value
Age(Year)	60.6 ± 10.2	53.8 ± 13.8	**0.001**
Gender(Male/Female)	18/38	118/159	0.147
Hypertension(Yes/No)	22/34	51/226	**0.001**
Diabetes mellitus(Yes/No)	10/46	29/248	0.117
Diagnosis(n)			0.972
LDH and/or LSS	47	233	
Spondylolisthesis	9	44	

Table 4 presented diagnostic characteristics of patients. Univariate analysis illustrated that the distribution of lower extremity symptoms might be related to the occurrence of FBSS. Patients without IC were less likely to develop FBSS (17.3% vs 60.7%, *P* < 0.05). Compared with the FBSS- group, the preoperative NRS-leg was significantly lower in patients with FBSS (6.3 ± 2.7 vs 7.3 ± 2.2, *P* < 0.05).

**Table 4:** Diagnostic characteristics

	FBSS + (*n* = 56)	FBSS-(*n* = 277)	*P* value
Symptom location(n)			**0.03**
Low back pain without limb symptoms	3	6	
Unilateral limb symptom	31	201	
Bilateral limb symptom	22	70	
Symptom duration(Month)	70.64 ± 95.2	57.9 ± 70.1	0.766
Aggravation(Yes/No)	40/16	220/57	0.187
Claudication(n)			**< 0.001**
No Claudication	22	229	
Claudication distances > 100 m	12	28	
Claudication distances ≤ 100 m	22	20	
Analgesic application(Yes/No)	34/22	175/102	0.728
Pre-op NRS-Back pain	6.1 ± 3.1	6.3 ± 2.8	0.836
Pre-op NRS-Leg pain	6.3 ± 2.7	7.3 ± 2.2	**0.005**

Comparison of the FBSS incidence among different surgical techniques showed significant differences. Postoperative rehabilitation may have an effect on the incidence of FBSS (*P* < 0.05) as shown in Table 5. Table 6 showed that HIZ and MCs on MRI were more prevalent in the FBSS + group than those in the FBSS- group.

**Table 5:** Surgical data

	FBSS + (*n* = 56)	FBSS-(*n* = 277)	*P* value
Type of surgery			**0.031**
FD	1	27	
PLIF	18	119	
PLF	14	59	
PLIF + PLF	23	72	
Levels of surgery			0.098
1	26	165	
2	27	100	
3	3	12	
Surgery time (minutes)	151.2 ± 59.2	144.2 ± 53.7	0.602
Rehabilitation(Yes/No)	13/43	31/246	**0.015**

**Table 6:** Radiographic parameters

	FBSS + (*n* = 56)	FBSS-(*n* = 277)	*P* value
HIZ(Yes/No)	29/12	185/35	**0.041**
Modic changes(Yes/No)	16/25	190/87	** < 0.001**
Pre-op LL (°)	31.1 ± 13.1	31.7 ± 13.9	0.608
Pre-op SL (°)	15.9 ± 9.2	15.5 ± 9.4	0.951

**Fig. 2:**
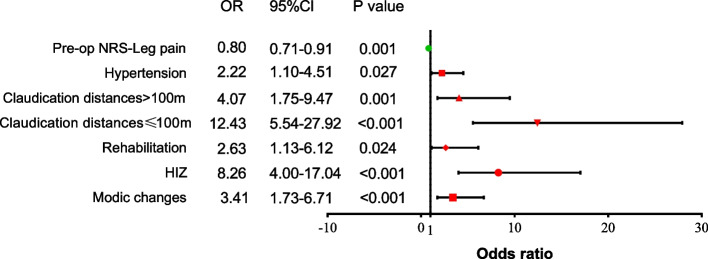
Multivariable logistic regression analysis: independent risk factors of FBSS

Results of the multivariable logistic regression analysis are shown in Fig. 2. A lower preoperative NRS-leg (OR:0.80, 95%CI:0.71–0.91, P = 0.001), hypertension (OR: 2.22, 95%CI: 1.10–4.51, *P* = 0.027), intermittent claudication with waking distance > 100 m (OR: 4.07, 95%CI: 1.75–9.47, *P* = 0.001), intermittent claudication with waking distance ≤ 100 m (OR: 12.43, 95%CI: 5.54–27.92, *P* < 0.001), HIZ (OR: 8.26, 95%CI: 4.00–17.04, *P* < 0.001) and MCs (OR: 3.41, 95%CI: 1.73–6.71, *P* < 0.001) on MRI, postoperative rehabilitation (OR: 2.63, 95%CI: 1.13–6.12, *P* = 0.024) were the independent risk factors of FBSS.

## Discussion

Failed back surgery syndrome (FBSS) was controversial since it was proposed in the 1970s because of its various definitions and diagnostic criteria [20, 21].The definition of ‘dissatisfaction’ and ‘persistent pain’ was equated simplistically and misused. In fact, patients with optimistic expectations for surgery had higher degree of satisfaction in despite of similar postoperative pain scores [22], which highlighted the necessity of our modification. The present study introduced a quantitative satisfaction evaluation in order to standardize the definition of FBSS.

The prevalence of FBSS ranges from 10 to 40% according to different researches [12, 23]. Persistent pain, frequent hospitalization and the resulting heavy financial burden will not only aggravate the doctor-patient contradictions, but also lead to an excessive occupation of medical resources [24]. Unfortunately, most of the existing studies focused on the psychological factors of patients, while ignoring the inherent characteristics of DLD [15, 16, 21]. This research, on the other hand, based on the comprehensive analysis of disease characteristics, identified a series of independent risk factors of FBSS in order to arouse the vigilance of clinicians and patients, so as to manage high-risk population effectively and promptly. Surgeries performed by six different senior spine surgeons improved the external validity of our outcomes.

Studies conducted in different medical centers have proved that hypertension has an adverse effect on chronic pain [25, 26]. What’s more, the intake of antihypertensive agents may increase pain sensitivity[27]. Hypertension is significantly associated with postoperative dissatisfaction for adult spinal deformities according to a multicenter retrospective study [28]. We speculate that this effect may be related to hypertension-mediated sympathetic nervous system dysfunction, which leads to a significant rise in neurological complications following operation [29]. At the same time, chronic pain caused by DLD plays an important role in blood pressure regulation by compromising activity in the descending pathways originating in brainstem regions that exert inhibitory influences on spinal neuronal function. The dysregulation of descending inhibition reduces the sensitivity of baroreceptors, which result in the impairment of cardiovascular regulation function with a concomitant increase in blood pressure. A vicious circle of ‘pain-hypertension-pain’ was formed eventually [30–32].

The current study confirms the negative effect of IC on spinal surgery. Based on five-year follow-up, a retrospective study noted that walking distance was significantly correlated with postoperative satisfaction of patients with DLD [33]. Sigmundsson et al. [34] analyzed 5100 patients collected prospectively and found that the rate of satisfaction reported by patients with walking distance > 1000 m was 2.4 times higher than that of patients with walking distance < 100 m. Similarly, a prospective study [35]reported that the risk of postoperative dissatisfaction could be increased by 10.3 times under the condition of walking difficulty who also showed a significant correlation with symptoms that may bring about FBSS, such as postoperative back pain, leg pain and numbness.

In the context of the rampant pandemic, NRS, whose reliability had been fully corroborated, is favored by clinicians due to its comprehensibility and higher compliance [36]. A retrospective cohort study [18] indicated that preoperative NRS leg pain was the only predictor of patient satisfaction following TLIF, which is analogous to our conclusion: for every 1-point increase in preoperative NRS-leg, the risk of FBSS is reduced by 20%. On the one hand, the operation leads to greater improvement in patients afflicted to more severe limb pain and result in increased satisfaction [37]. On the other hand, patients with lower NRS leg pain have more complicated operation willingness than those with higher NRS because of relatively mild neurologic symptoms. When patients with prolonged illness have to resort to surgery, whom they tended to regard as ‘the final solution’, holding too high or even unrealistic expectations that fully restore to health or return to work immediate postoperatively would finally result in dissatisfaction, even if the operation did improve neurological function to some extent.

HIZ and MCs, the reliable biomarkers of persistent pain, play an important role in the course of DLD by inducing inflammatory response [38, 39]. Preoperative MCs suggests poor clinical improvement and slow recovery [40], while HIZ indicates severe disc degeneration/displacement and the resulting severe, prolonged low back pain [41]. In addition, there were 5/12 HIZ and 8/25 MCs located outside the surgical segment, despite the lack of statistical significance, this might be another reason for refractory or even aggravated pain/numbness and consequent dissatisfaction of FBSS patients.

Contrary to a general impression, our result confirms that admission for rehabilitation treatment is a risk factor for FBSS. In this study, only 44 people were hospitalized for rehabilitation postoperatively, accounting for 13.2% of the total. Most of patients said it was out of economic considerations, while others were skeptical of the treatment itself. Despite a lot of research, there is no definite conclusion about mode and timing of postoperative rehabilitation, and even the necessity of hospitalization for it [42, 43]. In fact, a number of RCTs and systematic reviews had pointed out the limited benefits of rehabilitation relative to self-management in terms of improving of pain, walking ability, return to work, working ability, satisfaction and amelioration of poor surgical results [42, 44–46]. A similar effect of rehabilitation and sham treatment suggests that psychologic factors have a substantial effect on efficacy assessment [47]. In this case, the behavior of being hospitalized for further treatment implies the dissatisfaction with the effect of surgery, which can be further deepened with the extension of hospital stay and the increase of cost.

There are several limitations in this study. First, a relatively short follow-up time may mask changes in outcome indicators due to other degeneration in long-term follow-up. From another perspective, it enables us to rule out new symptoms caused by deterioration of the degeneration and use the above risk factors to identify risk groups efficiently. Besides, the conclusions derived from this single-center, retrospective study still needs to be verified by high-quality RCTs with a rigorous standard of diagnosis. Even so, we believe these risk factors can provide theoretical support for medical providers and encourage them to pay more attention to the management of high-risk groups.

## Conclusions

Open posterior lumbar surgery is an effective treatment for DLD which provides pain reduction and lumbar curve improvement with a considerable satisfaction rate. Lower preoperative NRS-leg, hypertension, intermittent claudication, HIZ, MCs and postoperative rehabilitation are risk factors for FBSS, which can serve as a tool for clinicians to identify at-risk population and provide more effective management to mitigate the doctor-patient contradictions and further occupation of medical resources.

## Data Availability

To avoid privacy being damaged from the research participants, the data will not be disclosed. Requests for data without being shown in this manuscript can be made to the corresponding author.

## Abbreviations

CT: Computer tomography
DLD: Degenerative lumbar disease
FBSS: Failed back surgery syndrome
FD: Fenestration discectomy
HIZ: High intensity zone
IC: Intermittent claudication
LBP: Low back pain
LDH: Lumbar disc herniation
LSS: Lumbar spinal stenosis
MCs: Modic changes
MRI: Magnetic resonance imaging
NRS: Numerical rating scale
NASS: North American Spine Society
PLIF: Posterior lumbar interbody fusion
PLF: Posterolateral fusion
RCT: Randomized controlled trial
